# Preliminary Study to Investigate Possible Cyto-Genotoxic and Oxidative Effects of Few-Layer Graphene in Human Bronchial Cells

**DOI:** 10.3390/ijms252413515

**Published:** 2024-12-17

**Authors:** Anna Maria Fresegna, Aureliano Ciervo, Cinzia Lucia Ursini, Raffaele Maiello, Francesca Tombolini, Valentina Del Frate, Marco Gentile, Delia Cavallo

**Affiliations:** Department of Occupational and Environmental Medicine, Epidemiology and Hygiene, Italian Workers’ Compensation Authority—INAIL, Via Fontana Candida 1, Monte Porzio Catone, 00078 Rome, Italy; c.ursini@inail.it (C.L.U.); r.maiello@inail.it (R.M.); f.tombolini@inail.it (F.T.); valentinadelfrate87@gmail.com (V.D.F.); ma.gentile@inail.it (M.G.); d.cavallo@inail.it (D.C.)

**Keywords:** graphene, graphene-based nanomaterials (GBNs), few-layer graphene (FLG), cytotoxic, genotoxic, oxidative, human lung cells

## Abstract

Graphene and its various derivatives, known as graphene-based nanomaterials (GBNs), hold tremendous potential across many fields due to their exceptional properties. As with any novel material, concerns about their safety have emerged alongside their widespread production and use. Several studies have shown that GBNs can have diverse effects on various cell lines and organisms under different exposure conditions. This study intends to evaluate the potential toxicity of few-layer graphene (FLG) in human bronchial BEAS-2B. Cells were exposed to different concentrations of FLG for 24 h, and the cyto-genotoxic, oxidative, and inflammatory effects were evaluated. We found an increase in cytotoxicity in terms of cell death, cell apoptosis, and membrane damage at the highest concentration. We also detected a slight increase in direct DNA damage and the percentage of comets. Oxidative DNA damage was observed at the highest concentration. FLG exposure did not induce notable cytokine release. Overall, this study suggests that exposure to FLG can induce cytotoxicity, apoptosis, and DNA damage in BEAS-2B, particularly at high concentrations. These findings contribute to a better understanding of the potential health effects of FLG and highlight the importance of considering dose-dependent effects when assessing the safety of GBNs.

## 1. Introduction

Graphene is a new material; it was produced for the first time in 2004 [1]. It is an allotrope of graphite, from which it is derived, and consists of a monolayer of carbon atoms in hexagonal rings in a lattice honeycomb arrangement [2]. Nowadays, graphene is widely used, even in its nano form, due to its properties of lightness, strength, thinness, and flexibility, as well as optical and electronic properties, thermal conductivity, and huge surface area [1,3].

Nowadays, its fields of application range from the chemical industry, healthcare, electronic devices, and biomedical research [4] to solar cells, food packaging [3], biosensors [5], drug delivery, and tissue engineering [6].

Graphene-based nanomaterials (GBNs) exist as monolayer graphene, few-layer graphene (FLG), graphene oxide (GO), reduced graphene oxide (rGO), graphene quantum dots (GQDs), and graphene nanoflakes, nanosheets, nanoribbons, and so on, depending on the number of graphene layers, the average lateral size, and the carbon-to-oxygen ratio [4,7].

Like most next-generation nanomaterials, doubts about the possible toxic effects of graphene arose only after its ubiquitous production and diffusion.

Recent studies have shown the diverse effects of GBNs across different cell lines and organisms under different exposure conditions.

In vitro, graphene oxide (GO) causes an increased reactive oxygen species (ROS) content in rat myocardial cells (H9C2) [8] and ROS-dependent DNA damage in human retinal pigment epithelium cells (ARPE-19) [9], but no genotoxic effect on the Jurkat E6-I lymphoblastoid cell line [10]. In addition, GO causes lipid peroxidation and the disruption of mitochondrial homeostasis in cultured human neuroblastoma cells [11]. Finally, GO has adverse effects on human sperm through oxidative stress, leading to reduced sperm motility and viability, as well as a more abnormal sperm morphology and DNA fragmentation [5]. Long-term exposure to GO induces permanent non-reversible genotoxic damage in HaCaT epithelial cells [7].

Single-layer graphene (SLG) causes varied levels of genotoxicity in various cell lines (primary rat alveolar macrophages (AMs), MRC-5 lung fibroblasts, and NR8383 and RAW 264.7 cells) [12].

Graphene platelets (GPs) induce dose-dependent DNA damage in the human acute monocytic leukemia cell line (THP-1) [13].

In vivo, GO alters the physiological, behavioral, and biochemical parameters in polychaetes [14] and induces decreased body weight, delayed development, and shortened lifespan in flies due to ROS accumulation and oxidative stress [15]. In addition, GO induces oxidative stress and genotoxicity in a concentration- and time-dependent manner in earthworms [16] and increased ROS production and mitochondrial damage in mice [8].

Graphene nanoplatelets (GNPs) affect various developmental, morphological, and physiological parameters in Drosophila melanogaster larvae [17].

Among GBNs, FLG has attracted much interest due to its application in hydrogen storage and reinforced nanocomposites [18], as well as in regenerative medicine and tissue engineering [19]. As a result, this nanomaterial is in increasing demand, proportionally increasing the potential degree of occupational exposure.

Few-layer graphene is prepared by the exfoliation of graphite with a top-down approach mostly employing a chemical vapor deposition process (CVD) [20,21,22]; it is made up of stacked graphene flakes (up to a maximum of 10) [19].

There are few studies on this type of graphene. Burgum et al. [18] exposed human lung epithelial (16HBE14o-) cells to FLG engineered with or without functional groups for 24 h and evaluated its cytotoxicity and genotoxicity. They found that neutral FLG and amine FLG induced genotoxicity secondary to oxidative and mitochondrial stress. Using primary, non-transformed normal human bronchial epithelial (NHBE) cells as an alternative to the most commonly used human lung tumor cell line (A549), Frontiñan-Rubio et al. [23] found that FLG caused NHBE cells to die preferentially by physical damage rather than by programmed cell death. Furthermore, the A549 cells seemed to be more resistant than NHBE cells to the cytotoxic effects of FLG. In another study, Burgum et al. [24] exposed human-transformed type-I (TT1) alveolar epithelial cell monocultures, as well as co-cultures of TT1 and differentiated THP-1 monocytes, to both functionalized and non-functionalized FLG. They found that FLG induced genotoxicity in the monocultures by primary-indirect DNA damage, while the co-cultured cells showed a secondary mechanisms of DNA damage driven by oxidative stress. In a study on recovery from damage, HaCaT skin keratinocytes were exposed to high concentrations of FLG for 24 or 72 h. The partial recovery observed was linked to the internalization of the material into cells [25]. Fusco et al. [26] analyzed skin irritation using the SkinEthic™ Reconstructed human Epidermis (RhE) model (Episkin, Lyon, France), a fully differentiated three-dimensional epidermal tissue. They found no skin irritation after a single instance of acute exposure. Malanagahalli et al. [27] reported no significant cytotoxic effects or inflammation in mouse macrophages exposed to FLG for 24 h [28]. Recently, Sallustrau et al. [21] studied the potential presence and distribution of radioactively labelled FLG within lung cells isolated from mice, suggesting a long-term accumulation of the material in the lungs over one year. Furthermore, they found higher bio-persistence of FLG in cases of acute exposure.

The FLG used in this study was produced in the R&D laboratory where we performed a biomonitoring study [29] and its one-year follow-up on exposed workers [30]. Another group performed an environmental monitoring study with characterization of the material on filters collected on site in real time [31,32,33]. The above cited biomonitoring study still represents the only one available one on workers producing/handling graphene-based materials. This study revealed genotoxic and oxidative effects using a Formamido-pyrimidine DNA glycosylase (Fpg)-comet assay on lymphocytes and a Buccal Micronucleus Cytome (BMCyt) assay. For this reason, we decided to test in vitro the same produced/handled FLG, evaluating also possible genotoxic effects on human bronchial cells by an experimental model already used to test the cyto-genotoxic effects of other nanomaterials, including multi-wall carbon nanotubes (MWCNTs) [34,35,36,37,38]. The present study represents the only one available that evaluates in vitro the cyto-genotoxic and oxidative effects of the same material produced by workers involved in a biomonitoring study.

We chose FLG also in consideration of the scarcity of studies on the topic, since most studies are focused on the effects of graphene oxide and reduced graphene oxide [39]. Therefore, in this study, we exposed BEAS-2B cells to 2, 10, and 40 µg/mL of FLG for 24 h, and we evaluated the cyto-genotoxic and oxidative effects and the release of pro-inflammatory interleukins (IL), IL-6 and IL-8, to contribute to a more in-depth knowledge of the mechanisms by which this material exerts its possible toxic action on lung cells. 

## 2. Results

### 2.1. FLG Characterization

**Figure 1:**
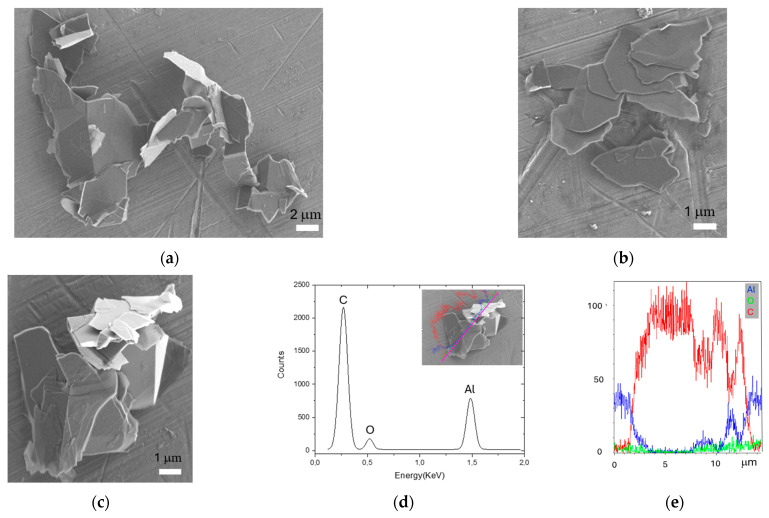
(**a**–**c**) FLG sample SEM images; (**d**) EdS sum spectrum acquired along the pink line (see inset) traced on the FLG aggregate (**c**) and the corresponding X-ray line profiles coming from carbon (red line), oxygen (green line), and Al (blue line) atoms (**e**).

A scanning electron microscopy analysis was carried out on the FLG sample. Figure 1 shows FLG aggregates composed of several flakes that have the same orientation, as shown in (b), or random orientation, as shown in panels (a) and (c). The largest lateral dimension of the aggregates in (a), (b), and (c) is below 20 µm; however, they are composed of flakes with smaller lateral sizes. By SEM morphological analysis, it was possible to estimate the diameters of the flakes, consistently differed from equivalent diameters based on a spherical approximation (i.e., aerodynamic diameter). From an occupational hygiene point of view, it is worth stressing that in the case of platelet-like shapes, as with FLG aggregates, for the same projected diameter, the smaller the thickness, the more the aerodynamic diameter differs from the projected diameter [33].

In Figure 1d, the energy dispersive x-ray spectroscopy (EDS) sum spectrum shows the X-ray signals coming from C, Al, and O atoms, that accumulate along the pink line depicted on the FLG sample (see inset panel (d)); moreover, in panel (e), the corresponding X-rays line profiles are shown.

In panel (d), it is possible to appreciate that the O contribution (green line) is low on the Al substrate (blue line), and it remains low on the sample, i.e., between 2 and 13 µm, on the pink line where the C contribution is the highest (red line). This highlights the fact that the aggregate shown in panel (c) consisted essentially of C atoms (i.e., oxygen free). Moreover, according to our SEM morphological analysis (panel (a) and panel (b)), the modulation of C X-rays profiles (d) indicated no uniform FLG thickness. The above-mentioned SEM and EDS characterizations of the sample agree with the technical specifications given by the producer.

### 2.2. FLG Dispersion

**Figure 2:**
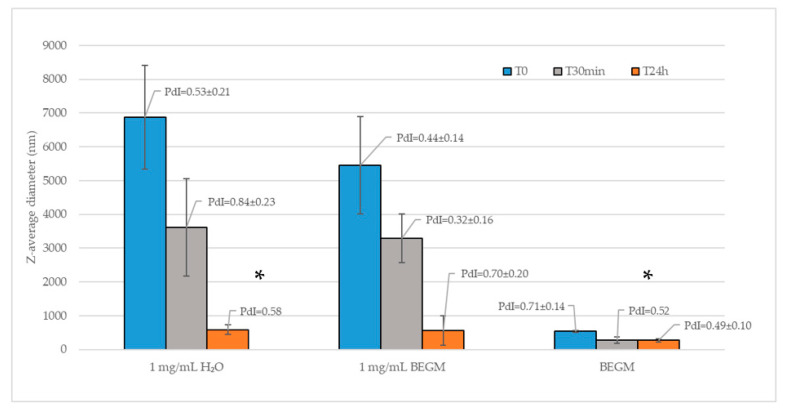
Comparison between media to dilute FLG, as highlighted by DLS analysis. Data represent the mean values of three independent experiments. The error bars represent the standard deviation of the data set. PdI (polydispersity index) mean values ± standard deviations are shown; *, *n* = 1.

We evaluated two media to disperse FLG: bronchial/tracheal epithelial cell growth medium (BEGM) and complete culture medium with the addition of retinoic acid and distilled water. The best turned out to be the BEGM, the hydrodynamic diameter (evaluated by dynamic light scattering (DLS)) was the smallest, indicating a better dispersion of the agglomerates/aggregates (Figure 2). Therefore, BEGM culture medium was used to dilute FLG for cell exposure.

**Figure 3:**
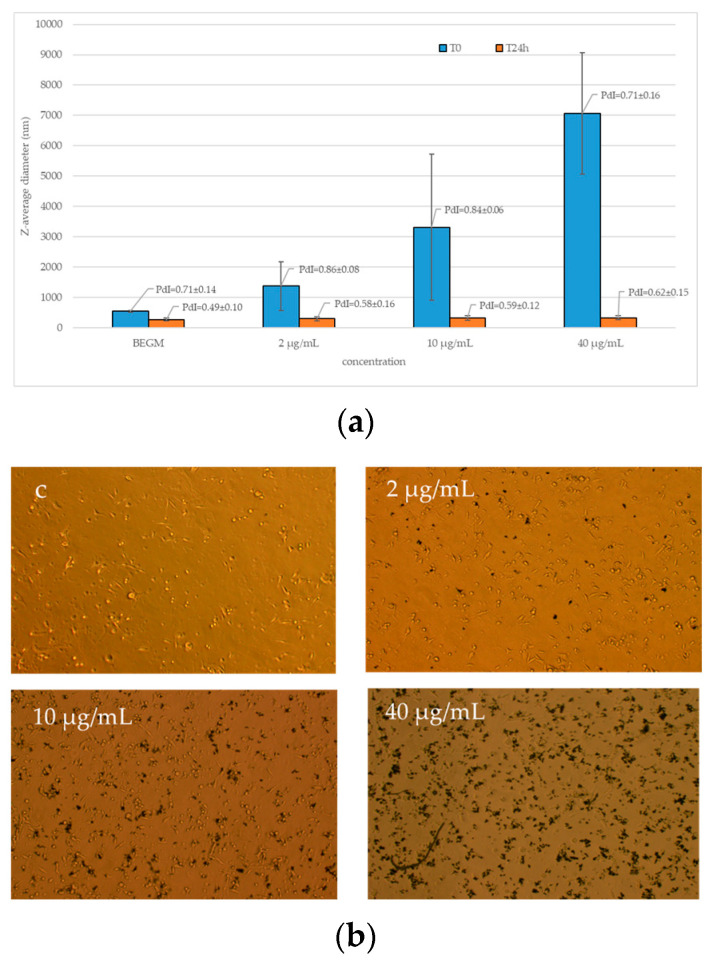
(**a**) DLS analysis of the size of agglomerates/aggregates of FLG in BEGM complete culture medium within 24 h. Data represent the mean values of three independent experiments. The error bars represent the standard deviation of the data set. PdI mean values ± standard deviations are shown. (**b**) Distribution of FLG on cultured cells after 24 h of exposure to final concentrations of 2, 10, and 40 µg/mL; c, negative control.

We also performed an evaluation of the size of the agglomerates/aggregates of FLG in BEGM complete culture medium within 24 h. All the agglomerates had precipitated after 24 h, resulting in the smaller hydrodynamic diameters at DLS analysis, and the powder was in contact with the cells at the bottom of the wells (Figure 3).

The Z potential of the 1 mg/mL solution in distilled water was −32.27 ± 11.5 (*n* = 3); in BEGM, it was −11.95 ± 1.34 (*n* = 2). Similarly, the Z potential of the 40 μg/mL solution in distilled water was −30.47 ± 18.21 (*n* = 3); in BEGM, it was −7.84 ± 2.96 (*n* = 3).

### 2.3. Cytotoxicity

**Figure 4:**
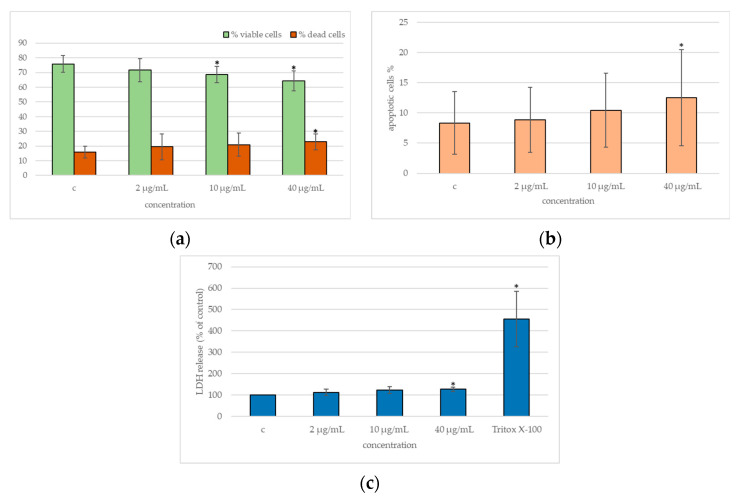
Cytotoxicity and apoptosis of BEAS-2B cells exposed to FLG for 24 h to final concentrations of 2, 10, and 40 µg/mL. (**a**) Viability–mortality and (**b**) apoptosis, expressed as percentages; cytofluorimetric assay by Guava EasyCyte Flow Cytometer; (**c**) Release of LDH expressed as a percent of control; LDH assay by cytotoxicity detection kit. c, negative control (unexposed cells); 1% Triton X-100, positive control. In all graphs, data represent the mean values of three independent experiments. The error bars represent the standard deviation of the data set. *p*-value: * ≤0.05 (Student’s *T* test).

Figure 4a,b shows the results of a cytofluorimetric assay. There was a slight decrease in cell viability at 10 and 40 µg/mL (a), whereas cell apoptosis increased in a statistically significant way only at the highest concentration, i.e., 40 µg/mL (b). This figure also illustrates the results of the lactate dehydrogenase (LDH) assay (c). We found a statistically significant increase of LDH release only at 40 µg/mL compared to unexposed cells, indicating the induction of membrane damage only at the highest concentration.

### 2.4. DNA Damage

**Figure 5:**
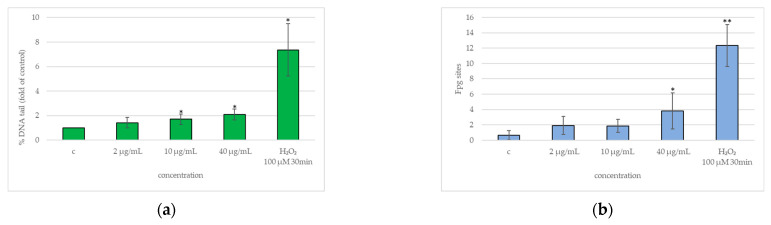

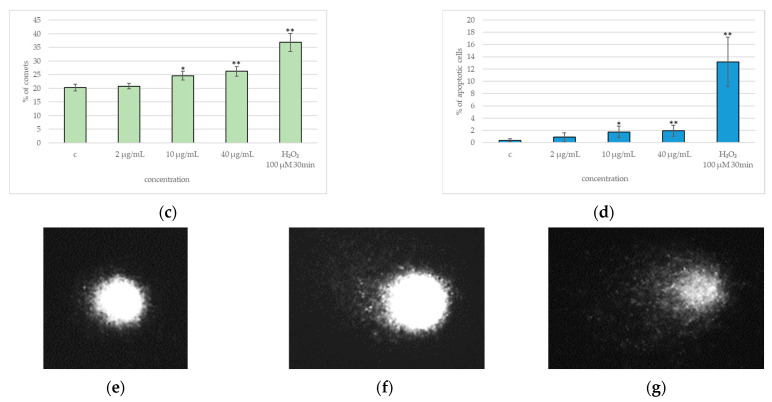
Fpg-Comet assay: (**a**) direct DNA damage (ratio of % tail DNA of exposed cells to % tail DNA of negative control), (**b**) oxidative DNA damage (% tail DNA subtracted from % tail DNA enz), (**c**) % of comets (% of comets out of total cells) and (**d**) % of apoptotic cells (% of apoptotic cells out of total cells) evaluation in BEAS-2B cells exposed to FLG for 24 h to final concentrations of 2, 10, and 40 µg/mL; c, negative control (unexposed cells); H_2_O_2_ (100 µM for 30 min), positive control. The results are expressed as means of three independent experiments. The error bars represent the standard deviation of the data set. *p*-values: * ≤0.05, ** ≤0.01 (Student’s *T* test). (**e**–**g**) typical images of comets with increasing DNA damage.

In Figure 5a, a slight but statistically significant increase in direct DNA damage (a), % of comets (c), and apoptotic cells (d) may be observed at 10 and 40 µg/mL; (b) the same trend was observed for oxidative damage to DNA, even if it was statistically significant only at the highest concentration of 40 µg/mL. The same figure shows typical images of comets with increasing DNA damage ((e), (f), and (g)).

### 2.5. Cytokines Release

**Figure 6:**
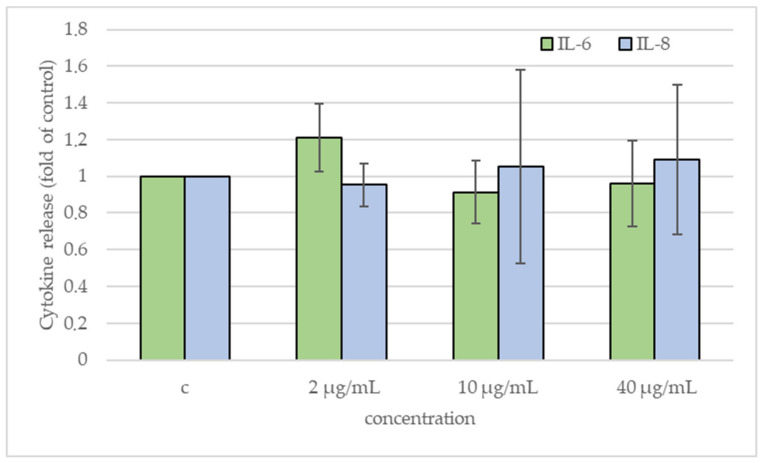
Cytokine release by ELISA. IL-6 and IL-8 release expressed as fold value of control of BEAS-2B cells exposed to FLG for 24 h to final concentrations of 2, 10, and 40 µg/mL; c, negative control (unexposed cells). The results are expressed as means of three independent experiments. The error bars represent the standard deviation of the data set.

There was no significant release of IL-6 and IL-8 after 24 h exposure, as an ELISA test showed (Figure 6).

## 3. Discussion

Research on the toxicity of few-layer graphene (FLG) is still ongoing, and the mechanisms are not fully understood. This work focuses on the study of possible cyto-genotoxic and oxidative effects and the release of pro-inflammatory IL-6 and IL-8 on BEAS-2B due to FLG in order to explore its mechanisms of action.

One of the bodily systems that comes into direct contact with materials we inhale is the respiratory system. Studies on this interaction can be very important for the development of safe-by-design protocols for the use and manipulation of these materials, for an adequate toxicity assessment, and to establish occupational exposure limits. In addition, research should be carried out using standardized criteria regarding the choice of the right biological model, mode of exposure, doses, and timing, in order to allow comparisons among studies. The lungs are also at risk when using a route other than the respiratory route to test the biodistribution of graphene in vivo. Several studies have shown that graphene administered orally, intravenously, or intraperitoneally may be found in the lungs [23].

Most published studies on this topic concern the effects of graphene in the lungs, so we decided to use BEAS-2B, a cell model that we found useful/suitable to investigate the potential toxicity of nanomaterials, such as pristine and functionalized multi-wall carbon nanotubes (MWCNTs) [34,36], TiO_2_ [35,37], and Co_3_O_4_ [38] on the respiratory tract.

The in vitro approach offers several advantages. First, it provides a controlled environment to expose human-derived cells to the materials under study, mimicking potential occupational exposure scenarios. This allows for a more relevant assessment of risks to human health. In addition, in vitro studies can be conducted with small sample sizes, reducing costs and minimizing the use of animals. The latter objective is part of research ethics, which emphasizes the importance of alternatives to animal testing, promoting practices in line with the principles of the 3Rs (replacement, reduction, refinement).

Our first attention was focused on the correct dispersion of FLG in different media in order to reduce, as much as possible, the size of any aggregates/agglomerates and to ensure the most uniform distribution of the material during the subsequent in vitro exposure of the cells. In fact, the right dispersion of materials is a crucial aspect in nanotoxicology. Controlling dispersion quality, by means of sonication, surfactants, and proper sample preparation, ensures accurate physicochemical characterization (particle size, size distribution, shape, aggregation/agglomeration, surface charge, etc.) and reliable toxicological assessments [40].

Combining several in vitro tests to assay cytotoxicity, genotoxicity, oxidative DNA damage, and inflammation provides a comprehensive overview of the main mechanisms involved in the toxicity of new materials with yet unknown effects [12].

Cytotoxic effects can manifest through mechanical damage to the cell membrane, leading to the release of lactate dehydrogenase (LDH), or through other forms of cellular damage, such as oxidative stress or the induction of apoptosis, ultimately resulting in increased cell mortality.

We detected a reduction in viability, albeit a modest one, and an increase in LDH release and apoptosis. This is consistent with studies on few-layer graphene by Burgum et al. [24], which indicated a statistically significant reduction in viability of about 20% in TT1 alveolar epithelial cells, although, in the opinion of the authors, this was not considered biologically detrimental.

Several authors have confirmed cellular uptake by TEM imaging in different cell lines via endocytosis/phagocytosis [18,24,25,27,28]. Burgum et al. 2021 [18] confirmed FLGH cellular uptake in NHBE cells, representing an alternative to BEAS-2B cells. In some cases, instead, direct mechanical penetration through the cell membrane has been reported, with consequent disruption of the cytoskeletal organization inside the cell [41]. Probably, such a disruptive mechanism could explain the increase we found in the release of LDH, a consolidated marker of cytotoxicity in terms of membrane damage [13].

The apoptotic effect, assessed by flow cytometry and Fpg-comet assay, was demonstrated as early as 10 µg/mL. This particular result confirmed the ability of FLG to induce apoptosis in the human bronchial epithelial (NHBE) cells, as also found by Frontiñan-Rubio et al. [23].

Regarding genotoxicity, we detected early DNA damage by the Fpg-comet assay, starting from a very low concentration (10 µg/mL) of FLG, in agreement with the results of Burgum et al. [18]. Those authors found a genotoxic effect in terms of the induction of an increase in micronuclei, starting from 10 µg/mL and increasing in a dose-dependent manner up to 100 µg/mL in a monoculture of human lung epithelial (16HBE14o-) cells exposed to neutral FLG for 24 h. With the same technique, we also found oxidative DNA damage only at the highest tested concentration (40 µg/mL), confirming the potential induction of oxidative stress of graphene-based materials on the respiratory tract [42].

Finally, in the present study, exposure to FLG did not trigger any release of (pro)-inflammatory mediators (IL-6, IL-8) in the culture supernatants, assayed by sandwich ELISA, in mouse bone marrow derived macrophages, as also reported by Malanagahalli et al. [27]. The observed cyto-genotoxic damage, although statistically significant, may not be sufficient to trigger the release of cytokines, or the release may have been below the limit of detection (LOD) of the test (0.92 pg/mL for IL-6 and 2.0 pg/mL for IL-8).

As mentioned above, deepening the knowledge on the toxicological profiles of new substances placed on the global market helps us to strengthen the tools for risk assessments in the occupational scenarios in which such materials are produced/handled/used. The main human health risk of graphene-based materials is associated with occupational exposure [26] by inhalation during production, use, and waste disposal [43]. Currently, our two studies on workers potentially exposed to FLG are the only ones available [29,30]. When comparing the present in vitro results with those obtained on workers exposed to the same graphene [29,30], it is possible to strengthen the relevance and reliability of the results. Indeed, the oxidative DNA damage and the apoptotic effects observed in BEAS-2B cells are in line with biomonitoring data, indicating consistency between in vitro and real-world exposure scenarios.

In conclusion, although FLG shows great promise for numerous applications, including biomedical applications, further research is needed to fully understand its potential toxicity and to develop safe and effective strategies for its use. In addition, comprehensive toxicological assessments, including long-term studies and risk assessments, will be essential for regulatory decision-making.

## 4. Materials and Methods

### 4.1. Characterization

The FLG nanoflakes used in this study were produced by a R&D laboratory using liquid phase exfoliation (LPE) from pristine graphite by wet-jet mill [44]. According to the technical specifications of the production laboratory, the flakes had an average lateral size of about 1.15 µm and a thickness of 1.6 nm.

We used a field emission scanning microscope (FegSEM, Zeiss, Oberkochen, Germany) equipped with an energy dispersive x-ray spectroscopy unit (EDS, Oxford Instrument INCA, Oxford, UK); the secondary electron signal, in high resolution mode, was acquired at EHT = 2 KV and at a working distance of 5 mm. The sample was dispersed in 2-propanol and sonicated for 15 min. The suspension was drop-cast on a stub covered with an aluminum filter.

We prepared stock solutions (1 mg/mL) of FLG in complete cell medium (BEGM, bronchial/tracheal epithelial cell growth medium with the addition of retinoic acid (Cell Applications, Inc., San Diego, CA, USA) in water to evaluate the dispersion of the material in different media. FLG was dimensionally characterized (hydrodynamic diameter) by dynamic light scattering (DLS) (Zetasizer nano ZS, Malvern, UK), at the beginning of exposure (t0) and after 24 h (t24), to evaluate suspension stability. The surface charge (Z potential) in distilled water or culture media was analyzed by electrophoretic light scattering (ELS) using the same device.

### 4.2. Cell Culture

Bronchial epithelial cells (BEAS-2B) were bought from the American Type Culture Collection (ATCC) (Rockville, MD, USA) and were cultured in complete BEGM. The cells, on the third passage, were seeded (8 × 10^4^ cells/well) into a 24 multi-well culture plate (15.6 mm well diameter) and cultured at 37 °C and 5% CO_2_ for 48 h before exposure. On the day of exposure, the cell medium was replaced with fresh medium.

### 4.3. Exposure

At the time of exposure, the stock solution in BEGM (1 mg/mL) was vortexed for a brief time, then sonicated for 30 min (Branson 2510; Branson Ultrasonics Corporation, Danbury, CT, USA) and vortexed again to better disperse the FLG. Then, a working solution (2, 10, or 40 µg/mL) in BEGM was prepared and quickly added to the wells. During exposure, cells were kept at 37 °C in 5% CO_2_ and humidified atmosphere. Three independent experiments were conducted.

### 4.4. Viability and Apoptosis

We evaluated viable, apoptotic, and dead cell percentages after 24 h exposure to FLG by cytofluorimetric assay, using the Guava EasyCyte Flow Cytometer (Merk Millipore, Darmstadt, Germany) and following the manufacturer’s directions. After exposure, the detached cells were mixed with the Guava ViaCount Reagent and left to incubate for 5 minutes at room temperature. A stained negative control (unexposed cells) was used to adjust the settings. A total of 1000 events were acquired. Thresholds and markers were adjusted to appropriately discriminate debris from cells, as well as live cells from dead and apoptotic cells. The data are presented as the mean value of three independent experiments.

### 4.5. Membrane Damage

To determine the membrane damage of cells exposed for 24 h to FLG, a lactate dehydrogenase (LDH) assay was performed (cytotoxicity detection kit; Roche Diagnostics, Mannheim, Germany). The LDH enzyme is present in all cells and is released into the supernatant in case of cell membrane damage. The assay is colorimetric and based on the reduction of tetrazolium salt to formazan by the LDH.

According to the manufacturer’s instructions, the reaction mixture plus the culture supernatant (1:1 ratio) were transferred in triplicate to the wells of an optically clear 96-well flat bottom plate and incubated for 30 min at T amb (15–25 °C), avoiding exposing the plate to light. Blank (only culture medium), negative control (cells not exposed), and positive control wells (cells exposed for 24 h to 1% Triton X-100) (Sigma-Aldrich, St. Louis, MO, USA) were included in each plate. At the end of incubation, a spectrophotometric microplate reader (iMark; Bio-Rad, Milan, Italy) was used to measure the absorbance at 490 nm.

After subtracting blank absorbance, the percentage of cytotoxicity was calculated as follows:

% cytotoxicity = (sample − negative control)/(positive control − negative control) × 100.

We did not find any interference in the light absorption due to FLG. A parallel set of experiments was conducted without cells; then, the absorbance of each concentration of FLG (without cells) was subtracted from that of the corresponding sample (with cells).

### 4.6. Genotoxic/Oxidative DNA Damage

Genotoxic/oxidative DNA damage was assessed by Fpg (formamido-pyrimidine DNA glycosylase; Sigma-Aldrich, St. Louis, MI, USA) modified comet assay. Direct and oxidative DNA damage, comets, and apoptotic cells percentage were evaluated.

After exposure, the cells were washed three times and then detached with trypsin (Sigma-Aldrich, St. Louis, MI, USA). Unexposed cells were used as a negative control, and cells exposed for 30 min to 100 µM H_2_O_2_ (Sigma-Aldrich, St. Louis, MI, USA) were used as a positive control. The cell suspensions from each experimental point were mixed with agarose and dispensed on two cut-to-size Gelbond films (Sigma-Aldrich, St. Louis, MI, USA) and placed on two microscope slides. One slide was treated with the enzyme Fpg (direct + oxidative damage), and the other was not (direct damage). This was followed by lysis (pH 10) and electrophoresis in alkaline buffer (pH 13) to detect DNA damage, as the damaged DNA strands migrated toward the anode and took the shape of a comet upon observation. After washing in Tris-HCl 0.4 M (Corning, Mediatech, Inc., Manassas, VA, USA), the slides were stained with ethidium bromide 20 µg/mL (Sigma-Aldrich, St. Louis, MI, USA). We randomly took images of 100 comets per slide using a fluorescence microscope (Axioplan 2 Imaging, Carl Zeiss, Göttingen, Germany) and then analyzed them with a specific image analysis software (Delta Sistemi, Rome, Italy).

We calculated the mean % tail DNA of comets from enzyme untreated cells (% tail DNA) for direct DNA damage and the mean % tail DNA of comets from Fpg-enzyme treated cells (% tail DNA enz) for direct and oxidative DNA damage at each experimental point. Then, direct DNA damage was computed as the ratio of % tail DNA of exposed cells to % tail DNA of the negative control. Oxidative DNA damage (Fpg-sensitive sites) was calculated by subtracting % tail DNA from % tail DNA enz. % of comets and % of apoptotic (extremely DNA damaged cells, showing a very small head of the comet and the most of DNA in the tail) cells [45] and is expressed as a percentage of the total cells analyzed. The results are expressed as means of three independent experiments.

### 4.7. Cytokines

The release of IL-6 and IL-8 after 24 h exposure to FLG was quantified by Enzyme-Linked ImmunoSorbent Assay (ELISA) (Human Platinum ELISA corresponding kit, eBioscience, Wien, Austria), according to the manufacturer’s guidelines. Briefly, the cellular supernatant was placed in contact with the kit microwells coated with specific interleukin capture antibodies. A detection antibody was added to the wells to also bind to the same interleukin (sandwich). After several washing steps and incubations, an enzyme and its substrate bound to the sandwich of antibodies to make it visible. The amount of colored product is directly proportional to the amount of interleukin in the well. The limit of detection (LOD) is 0.92 pg/mL for IL-6 and 2.0 pg/mL for IL-8 ELISA Kit. The cell supernatant was collected and stored at −20 °C until use. Samples, negative controls (cells not exposed to FLG), proper cytokine standards, and blank and control samples were assessed in duplicate. We quantified the absorbance at 450 nm by a microplate absorbance reader (iMark; Bio-Rad, Milan, Italy).

### 4.8. Statistical Analysis

We performed three independent experiments for each assay and expressed the data as mean ± standard deviation (SD). We analyzed the statistical significance of the difference between exposed and unexposed cells by paired parametric two-tailed Student’s *t* test (Microsoft Office Excel) and considered *p*-values of ≤0.05 and ≤0.01 as statistically significant.

## 5. Conclusions

In summary, under our experimental conditions, FLG nanoflakes induced a slight dose-dependent cyto-genotoxic and oxidative effect in BEAS-2B cells, with statistically significant effects observed only at the highest concentration.

Given the huge amount of new graphene-based products and the speed with which they are being produced, the scientific community will have to work on better evaluating the potential negative effects of FLG nanoflakes on human health and the environment, even before these substances are placed on the market, to guarantee safe-by-design production.

## Data Availability

The data presented in this study are available within this article. Further inquiries may be directed to the authors.
